# Structural Elucidation of Novel Stable and Reactive Metabolites of Green Tea Catechins and Alkyl Gallates by LC-MS/MS

**DOI:** 10.3390/antiox11091635

**Published:** 2022-08-23

**Authors:** Ons Ousji, Lekha Sleno

**Affiliations:** Chemistry Department, Université du Québec à Montréal, Downtown Station, P.O. Box 8888, Montréal, QC H3C 3P8, Canada

**Keywords:** green tea catechins, EGCG, synthetic gallates, antioxidants, metabolites, in vitro incubations, liquid chromatography, high-resolution tandem mass spectrometry

## Abstract

Synthetic gallic acid derivatives are employed as additives in food, personal care products, and pharmaceutical formulations. Despite their widespread use, little is known about their human exposure, health effects, and metabolism. Green tea catechins are natural antioxidants, known for their health-promoting properties, and are also employed as food additives or in personal care products. The objective of this study was to establish metabolic pathways involved in the biotransformation of green tea catechins and synthetic gallate esters. Liquid chromatography coupled with high-resolution tandem mass spectrometry (LC-HRMS/MS) was used to elucidate oxidative and methylated metabolites, in addition to glutathione conjugates, formed in vitro using human liver microsomal incubations. The developed method was applied to 14 different parent compounds with a wide range of polarities, for the structural elucidation of many known and novel metabolites. These results serve to inform about the wide variety of possible metabolites formed upon exposure to these compounds.

## 1. Introduction

Lipid peroxidation is the primary cause of the formation of undesired byproducts in food, cosmetics, pharmaceuticals, and petrochemicals [1]. The addition of antioxidants is commonly used to inhibit or slow this process, to increase shelf life, and to maintain freshness and texture of consumer products [2,3]. These compounds can inhibit free radical formation and thus interrupt autoxidation [1]. Synthetic antioxidants present many advantages, including low cost, ease of synthesis, and high efficiency [4,5]. However, as a result of the global movement toward using natural ingredients in food products, medications, and personal care products, natural antioxidants are gaining interest [6]. These antioxidants include vitamins (C and E), herbs and spices, and natural products from plants [1,6]. They are used as preservatives and bioactive molecules for prevention against diseases such as cancer [7], obesity [8], neurological disorders [9], aging [10], and cardiovascular diseases [11]. They are often considered a healthier alternative to synthetic antioxidants [3].

Plant extracts such as green and black teas have been widely studied for their antioxidant activities [10]. Green tea extracts have the highest total phenolic content, 94% of which are flavonoids (catechins) and phenolic acids (e.g., gallic acid (GA)) [1,4]. Flavonoids consist of a flavan-3-ol unit, including catechin (C), epicatechin (EC), gallocatechin (GC), and epigallocatechin (EGC), in addition to galloylated catechins, in the case of catechin gallate (CG), epicatechin gallate (ECG), gallocatechin gallate (GCG), and epigallocatechin gallate (EGCG) [12]. These tea polyphenols have gained special attention due to their numerous beneficial effects (antioxidant [13], antimicrobial [14], antiviral [15], and antifungal effects [16], in addition to protective effects against different cancers [12,17]). The relative effectiveness of different catechins is related to their structure (for example, the number of hydroxyl groups and their positions, and the accessibility of active groups), concentration, and stability [18].

The structure–activity relationships of tea polyphenols on cancer chemoprevention have been studied [12,19], indicating that the galloyl moiety is a specific structural feature for the chemopreventive [19], weight reducing [20], and potential anti-HIV [21] properties of catechins. Despite all these advantages, the development of catechins as natural antioxidants and therapeutic agents faces several challenges, including low bioavailability and rapid degradation [14]. Polyphenols consumed in the diet are readily accepted by consumers, even if they can present stability issues and lower antioxidant activity, thus needing to be consumed in larger amounts [5]. However, it is not clear what deleterious effects could be related to consuming them at very high concentrations.

These challenges related to natural polyphenols have inspired the synthesis of several gallate esters, such as ethyl gallate (EG), propyl gallate (PG), butyl gallate (BG), octyl gallate (OG), lauryl gallate (LG), hexadecyl gallate (HG), and octadecyl gallate (SG) [22]. These molecules differ by the length of their alkyl side chains, ranging from 2 to 18 carbons, thus having widely different polarities. GA alkyl esters with medium chain lengths have demonstrated very high antioxidant activities and the hydrophobic moiety contributes to the increased bioavailability of these compounds [22,23]. These molecules are primarily used as food additives and antibacterial agents [23]. Some studies have supported their classification as “healthy” ingredients [23], while in contract, their cytotoxicity has been demonstrated in rat hepatocytes [24].

The data available on the biotransformation of tea catechins and synthetic gallate esters is limited and studies have concentrated mainly on EGCG or several of its analogs [25]. The major biotransformation reactions described in literature for tea catechins include methylation, glucuronidation, and sulfation [26,27]. Methylation is the primary metabolic route for these molecules performed by the enzyme catechol-*O*-methyltransferase (COMT) [26]. The occurrence of methylated metabolites of tea catechins has been reported [26,28,29]. Crespy et al. [27] investigated the glucuronidation of some catechins by hepatic and intestinal microsomes. They identified four major glucuronide conjugates of EGCG and EGC by tandem mass spectrometry (MS/MS) and NMR spectroscopy. The sulfation of catechins via sulfotransferases has also been reported [30]. However, the formation of reactive metabolites of tea catechins and synthetic gallate esters via the detection of glutathione conjugates has not been extensively studied.

The in vitro metabolism of eight major green tea catechins (C, EC, GC, EGC, CG, ECG, GCG, and EGCG), gallic acid (GA), and five synthetic gallate esters (EG, PG, BG, OG, and LG) has been investigated using liquid chromatography coupled with a quadrupole-time-of-flight high-resolution tandem mass spectrometer. Oxidative metabolites, glutathione adducts and methylated metabolites of these 14 compounds were studied using human liver microsomal incubations. The developed analytical approach achieved the structural characterization of over 100 metabolites.

## 2. Materials and Methods

### 2.1. Chemicals

Green tea catechins [(+)catechin (C), (−)epicatechin (EC), (−)catechin gallate (CG), (−)epicatechin gallate (ECG), (−)gallocatechin (GC), (−)epigallocatechin (EGC), (−)epigallocatechin gallate (EGCG), (−)gallocatechin gallate (GCG)], synthetic gallate esters [ethyl gallate (EG), propyl gallate (PG), butyl gallate (BG), octyl gallate (OG) and lauryl gallate (LG)], gallic acid (GA), reduced *L*-glutathione (GSH), *S*-(5′-adenosyl)-*L*-methionine (SAM), nicotinamide adenine dinucleotide phosphate (NADP^+^), glucose-6-phosphate, magnesium chloride and glucose-6-phosphate dehydrogenase, potassium (mono- and di-basic) phosphate, in addition to HPLC-grade acetonitrile (ACN), methanol, and acetic acid were purchased from Sigma-Aldrich (Oakville, ON, Canada). Human liver microsomes (HLM, pooled from 50 donors) were purchased from Corning (Corning, NY, USA). Ultrapure water was obtained from a Millipore Synergy UV system (Billerica, MA, USA).

### 2.2. In Vitro Incubations

All catechins and galloylated compounds were incubated at 20 µM with human liver microsomes (1 mg/mL protein), a NADPH-regenerating system (5 mM MgCl_2_, 0.5 mM NADP^+^, 10 mM glucose-6-phosphate and 2 units/mL glucose-6-phosphate dehydrogenase), with and without 5 mM GSH and 1 mM SAM at 37 °C for 1 h in 100 mM phosphate buffer, at a pH of 7.4. Control samples were prepared without NADPH and/or without GSH or SAM. An equal volume of cold acetonitrile was added to quench the reaction, followed by centrifuging for 8 min at 14,000 rpm, at 4 °C. The supernatants were then evaporated and reconstituted in 10% acetonitrile back at original incubation concentrations prior to LC-MS/MS analysis.

### 2.3. LC-HRMS/MS Analysis and Data Processing

LC-MS/MS analysis were performed using a Shimadzu Nexera HPLC coupled to a Sciex 5600 TripleTOF^®^ (quadrupole-time-of-flight) system (Concord, ON, Canada), in negative ion electrospray mode, equipped with a DuoSpray source. Chromatographic separation was achieved using a Phenomenex Kinetex^®^ biphenyl (100 × 2.1 mm, 2.6 µm) column, with mobile phases of water (A) and methanol (B), both containing 0.1% acetic acid, at 0.25 mL/min and a column temperature of 40 °C. The injection volume was 25 µL. The HPLC gradient was as follows: 5% B held for 0.5 min, linearly increased to 50% at 15 min, up to 95% at 18 min, and held for an additional 3 min. For gallic acid incubations, a slower gradient was used as follows: 3% B held for 1 min, linearly increased to 30% at 15 min, up to 85% at 18 min.

Ion source parameters included ionization voltage of 5000 V, curtain gas of 35 psi, drying and nebulizer gases at 50 psi, source temperature of 450 °C, and declustering potential of 60 V. TOF-MS spectra were acquired (with 250 ms accumulation time), followed by MS/MS in information-dependent acquisition (IDA) mode on the 5 most intense ions using dynamic background subtraction (175 ms each). Targeted MS/MS analyses were performed when necessary for better spectral quality of metabolites which were not triggered by IDA method. Collision-induced dissociation was performed with 30 ± 10 V collision offset-voltage. Nitrogen was used as collision gas, and collision energy was 30 ± 10 V. MetabolitePilot 2.0 (Sciex) software was employed to screen samples for potential metabolites using a set of known biotransformation reactions, including oxidative reactions, GSH conjugation and methylation, and combinations thereof. PeakView 2.2 and MasterView 1.1 (Sciex) were also used for processing LC-MS/MS data to confirm and expand the list of detected metabolites based on mass accuracies (all within 5 ppm), isotope patterns, and MS/MS analyses.

## 3. Results

### 3.1. LC-MS Method Development and MS/MS Fragmentation of Catechins and Gallate Esters

**Figure 1:**
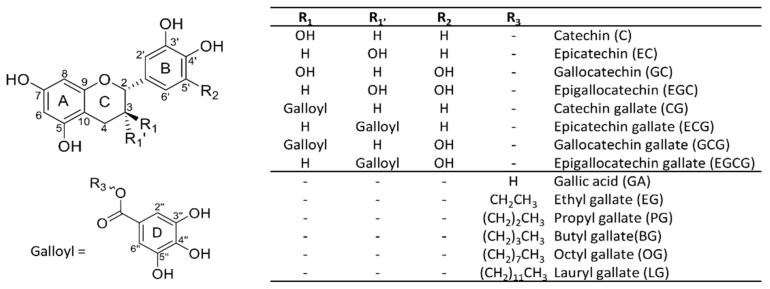
Chemical structures of studied natural and synthetic antioxidants.

The metabolism of eight green tea catechins, gallic acid and five analogs of synthetic gallate esters (Figure 1) was studied by LC-HRMS/MS using human liver microsomal incubations to form oxidative and methylated metabolites, in addition to glutathione conjugates.

**Figure 2:**
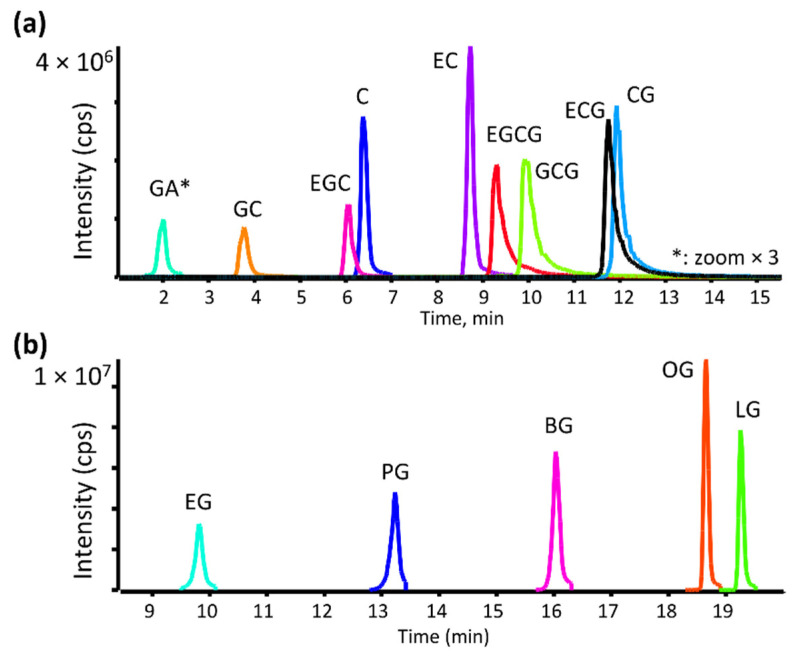
Overlaid LC-TOF-MS extracted ion chromatograms of deprotonated molecules from natural (**a**) and synthetic (**b**) antioxidants from control HLM incubations. GA peak (*) was increased by 3× for clarity.

A robust chromatographic method was developed to separate all studied compounds, a biphenyl column using water and methanol, both containing 0.1% acetic acid as mobile phases. This method allowed for good separation between all catechins, including epimeric species (Figure 2a) with increased resolution and shorter run times than reported previously [31]. The optimized gradient allowed the detection of highly polar gallic acid as well as the most hydrophobic lauryl gallate ester in one method (Figure 2b).

**Figure 3:**
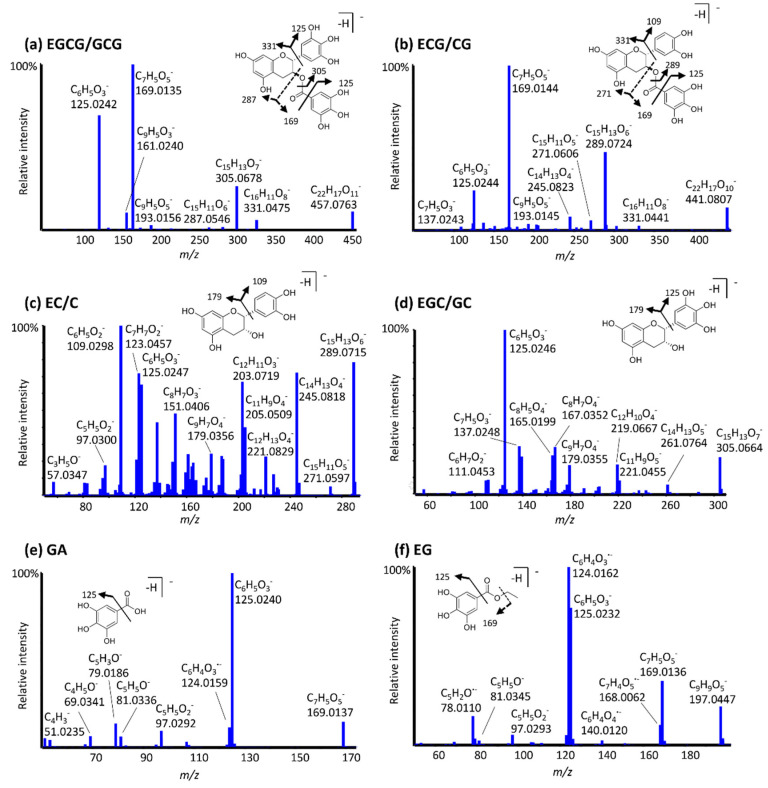
High-resolution MS/MS spectra for deprotonated EGCG/GCG (**a**), ECG/CG (**b**), EC/C (**c**), EGC/GC (**d**), GA (**e**), and EG (**f**).

The MS/MS spectra and fragmentation behavior of green tea catechins, gallic acid, and ethyl gallate are presented in Figure 3a–f. For the catechins, epimers indicated very similar fragmentation. Both EGCG and GCG, for example, fragment to form an ion at *m/z* 331.04, indicating the presence of an unmodified galloyl ester moiety, with a base peak at *m/z* 169.013, corresponding to deprotonated gallic acid. This product ion is also observed for ECG/CG, however, it is absent in spectra of catechins lacking the gallic acid ester function, such as EC/C and EGC/GC. ECG/CG spectra exhibit a fragment ion at m/z 289.07 corresponding to [M-H-152]^−^, following cleavage at the ester bond. EC and C exhibit different fragmentation from other catechins, with a base peak at *m/z* 109.03 corresponding to the C_6_H_5_O_2_^−^ ion. The ion *m/z* 125.02 is observed for all catechins, while its intensity varies from 100% for EGC/GC to 20% for EGCG/GCG. It is not affected by the presence of an additional phenolic hydroxyl group, or by the presence of the pyrogalloylated moiety. Similar results were observed by Miketova et al. [32] and this ion was proposed as the unmodified A ring [32]. Deprotonated gallic acid also forms a predominant peak at *m/z* 125.02 upon fragmentation, corresponding to the pyrogallol moiety. All gallate esters fragment similarly, with common peaks at *m/z* 169.01, 168.01, 125.02, 124.02, and 78.01, corresponding to loss of the side chain, C_7_H_4_O_5_^−^, pyrogallol moiety, C_6_H_4_O_3_^∙−^, and C_5_H_2_O^∙−^, respectively.

### 3.2. Metabolite Identification

The metabolism of major green tea catechins (C, EC, GC, EGC, CG, ECG, GCG, and EGCG), gallic acid (GA) and synthetic gallate esters (EG, PG, BG, OG, and LG) was studied to elucidate biotransformation reactions. Table 1 and Table 2 summarize the results for all detected metabolites of natural catechin antioxidants and gallic acid esters, respectively. More detailed information of these compounds and their metabolites, including MS/MS fragmentation results, can be found in Supplemental Information Tables S1 and S2.

#### 3.2.1. Oxidative Metabolites

Of all the tested compounds, only BG, OG, and LG produced hydroxylated metabolites, corresponding to two monohydroxylated isomers each for butyl, octyl, and lauryl gallate, in addition to a di-hydroxy octyl gallate (Table 2). All detected oxidative metabolites and parent compounds demonstrated common fragment ions at *m/z* 169.01, 125.02, and 124.01, from the intact gallic acid, the pyrogallol moiety, and radical ion C_6_H_4_O_3_^∙^^−^, respectively. These results together with the absence of *m/z* 185 prove that the oxidation is happening in the side chain and not in the gallic acid moiety. Under these conditions, OG and LG is also observed to form aldehyde metabolites. For example, the MS/MS spectrum of OG aldehyde (−2H+O) demonstrated a peak at 267.1227, corresponding to the loss of CO from the terminal carbon where the aldehyde is formed. OG and LG aldehydes can be further oxidized to the corresponding carboxylic acids (Figure S1). Both acids (−2H+2O) exhibit a loss of water and CO_2_ upon fragmentation, placing the carboxylic acid on the terminal methyl.

#### 3.2.2. Methylated Metabolites

**Figure 4:**
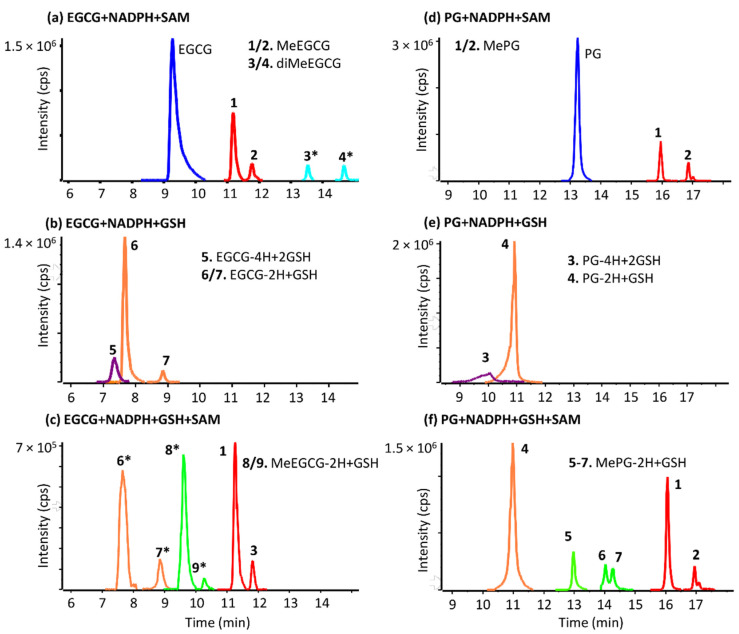
Overlaid extracted ion chromatograms of EGCG (**a**–**c**) and PG (**d**–**f**) metabolites formed in HLM incubations. Peaks with asterisk (*) were increased by 10× for clarity.

**Figure 5:**
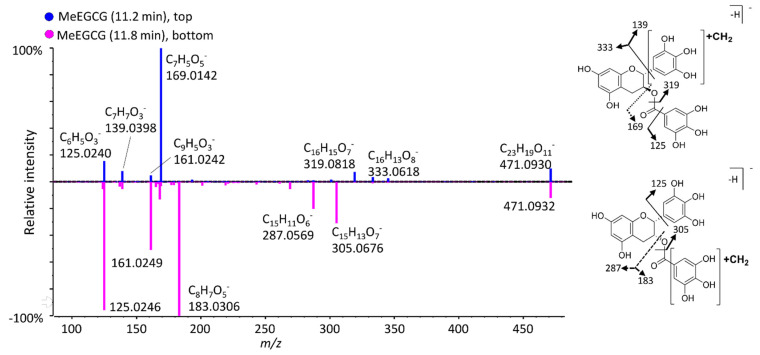
Mirror plot of MS/MS spectra for two MeEGCG isomers with proposed structures supported by diagnostic fragment ions.

When SAM was added to the incubations as a cofactor, all 14 studied compounds produced two mono-methylated metabolites (Figure 4, Table 1 and Table 2). For instance, both EC and C formed two mono-methylated isomers, having four different retention times, indicating four unique structures. In theory, these catechins can be either methylated on the A or B-ring, and their MS/MS fragmentation unfortunately does not allow us to differentiate between these two possibilities (Table S1). Conversely, the MS/MS spectra of both MeEGCG isomers (compared in Figure 5) allow us to pinpoint when the D ring is methylated. The isomer at 11.8 min has two unique fragment peaks at *m/z* 305.0676 and 183.0306, corresponding to C_15_H_13_O_7_^−^ and C_8_H_7_O_5_^−^ (methylated gallic acid), respectively, proving that the methyl is added on the D ring. The isomer at 11.2 min exhibits fragments at *m/z* 319.0818, 169.0142, and 139.0398, from C_16_H_15_O_7_^−^, deprotonated gallic acid and methylated pyrogallol, respectively, corresponding to a methylated A or B ring. Lu et al. [26] compared the methylation of EGCG and EGC in humans, mice, and rats, indicating that O-methylation occurs mainly at the 4′-position of the B-ring in EGC and the 4″-position (of the D-ring) in EGCG [26]. For catechins lacking a D-ring, such as EGC, two methylated isomers and one di-methylated metabolite were detected (Table 1). Comparison of their MS/MS fragmentation (Table S1) demonstrated that EGC can be methylated in the B-ring (isomer detected with higher intensity) and the A-ring. Meng et al. [33] identified one O-methylated EGC in human urine and blood samples and elucidated its structure as 4′-*O*-MeEGC by NMR.

As a representative example of gallate esters, in Figure 4, two well separated mono-methylated metabolites are seen for propyl gallate. Both isomers have identical MS/MS spectra, with ions at *m/z* 139.004 and 183.030, consistent with the added methyl on the pyrogallol group at two different positions. For gallic acid, one di-methylated metabolite was detected. For BG and OG, an oxidized methylated metabolite (+O+CH_2_) was also formed. All octyl gallate metabolites characterized in this work are represented in extracted ion chromatograms in Figure S1.

#### 3.2.3. GSH Adducts

**Figure 6:**
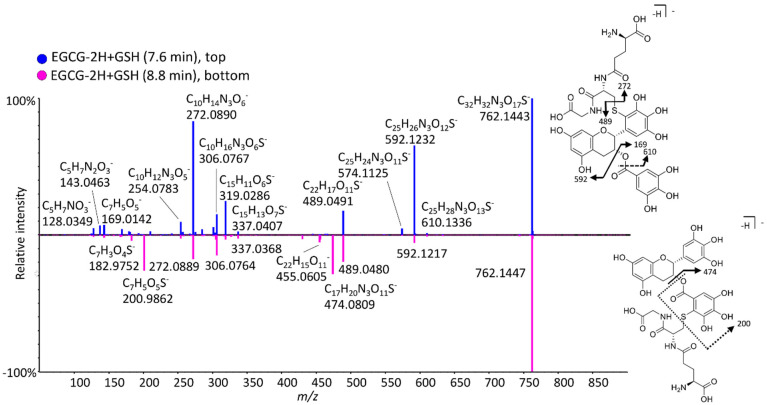
Mirror plot of MS/MS spectra for two EGCG-2H+GSH adducts with proposed fragmentation pathways for distinguishing structures.

GSH is commonly used as a trapping agent of unstable reactive metabolites, and GSH adducts were detected for all examined compounds (Table 1 and Table 2). When EGCG was investigated, (Figure 4) two glutathionylated isomers of EGCG-2H+GSH and a di-GSH adduct were detected. The MS/MS spectra of the two mono-glutathionylated adducts are compared in Figure 6. The adduct at 7.6 min forms fragment ions at *m/z* 169.0142, 592.1232, and 610.1336, corresponding to a gallic acid moiety, [M-H-170] (neutral loss of GA), and the ion C_25_H_28_N_3_O_13_S^−^(loss of C_7_H_5_O_4_), proving that the D-ring remains intact, and that GS binds to the B-ring. However, the isomer at 8.8 min has unique peaks at *m/z* 474.0809 and 200.9862, corresponding to GSH binding to the gallic acid moiety and the ion C_7_H_5_O_5_S^−^ (gallic acid with the sulfur from GSH still attached), respectively, therefore, glutathione binds to the D-ring. Muzolf-Panek et al. [34] previously identified 2′-glutathionyl-EGCG and 2′,6′-diglutathionyl-EGCG when EGCG was incubated on the presence of GSH and tyrosinase.

**Figure 7:**
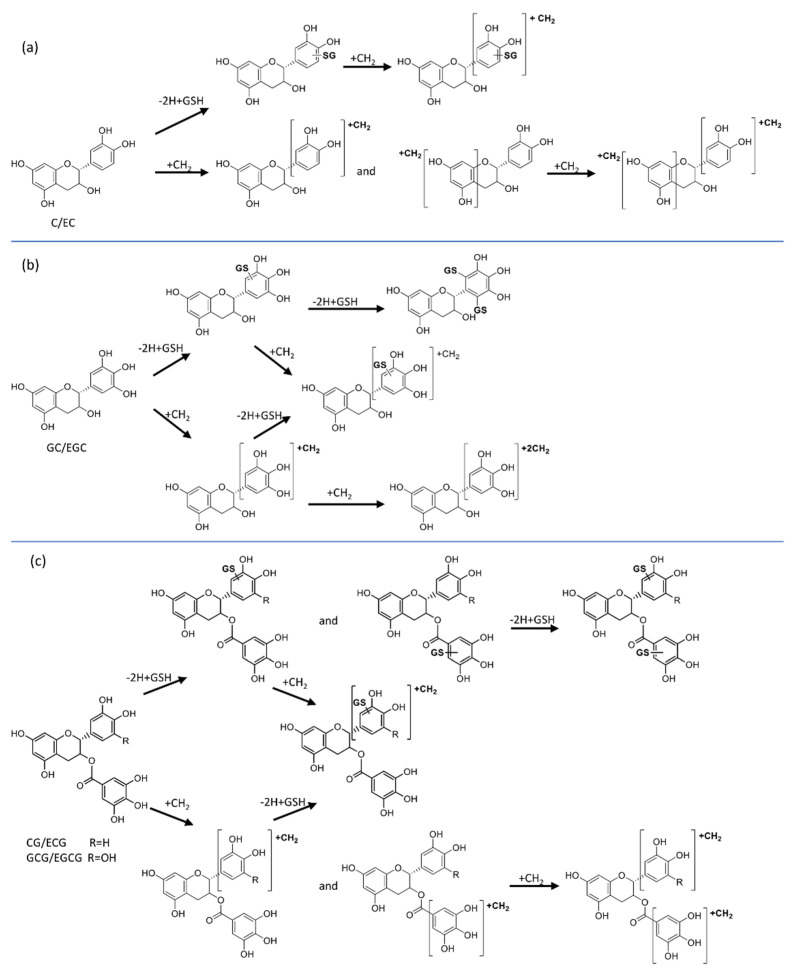
Proposed biotransformation pathways of catechins forming GSH adducts and methylated metabolites of (**a**) C/EC, (**b**) GC/EGC, and (**c**) CG/ECG and GCG/EGCG.

**Figure 8:**
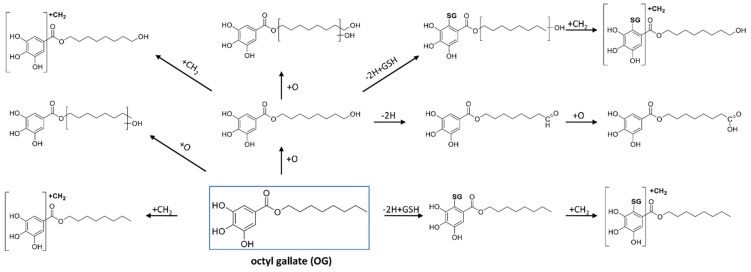
Proposed biotransformation pathways for the formation of metabolites and GSH adducts from octyl gallate.

The studied natural antioxidants containing a pyrogallol and/or a galloyl moiety (GC, EGC, CG, ECG, GCG, and EGCG) all produced at least two mono-GSH adducts in addition to their methylated forms (Table 1). All except catechin and epicatechin also produced di-GSH adducts. Oxidation of catechins forming a catechol in the B-ring leads to the formation of a corresponding reactive o-quinone that reacts with the thiol group in GSH [35]. The galloyl structure (having three hydroxy groups) on the B ring and the presence of the D ring increases the chance for the formation of a double GSH adduct. Two schemes summarizing the biotransformation pathways of catechins and of synthetic gallate are presented in Figure 7 and Figure 8, respectively.

Synthetic gallates also produced several GSH conjugates, including mono-GSH adducts in all cases, di-GSH adducts for PG and BG, and hydroxylated mono-GSH conjugates for OG and LG. For instance, PG produced one GSH adduct and one di-GSH conjugate and three methylated GSH adducts (Figure 4e,f). All detected conjugates formed common characteristic negative ion fragments from the GSH moiety (306.07, 272.08, 254.07, 210.08, 143.04, and 128.03) (Table S2) [36]. As a representative example, Figure 8 presents the biotransformation pathways of octyl gallate.

#### 3.2.4. Methylated GSH Adducts

All studied compounds produced GSH adducts of mono-methylated metabolites. EGCG produced two MeEGCG-2H+GSH isomers (Figure 4c). These two metabolites shared the fragment ion at *m/z* 169.013 (deprotonated gallic acid), demonstrating that GSH conjugation and methylation did not occur in the D-ring. All synthetic gallates produced methylated GSH adducts, with several isomers for many of them (Figure 8). PG produced three separated isomers of MePG-2H+GSH (Figure 4f). These three isomers had different retention times from BG-2H+GSH, despite the same neutral formula. The presence of three hydroxy groups on the galloyl side of PG that can be methylated and the binding of GSH could result in three different GSH conjugates methylated at different positions. This proves that methylation also occurs on the ring and not on the side chain. As the acyl chain increases in length, less methylation is seen.

## 4. Discussion

Since green tea is one of the most widely consumed beverages in the world [18] and tea catechins have important antioxidant and anticancer properties [7], it is imperative that we fully understand the potential metabolic pathways involved in the biotransformation of these substances. Once these metabolites are characterized, further studies can examine their individual bioactive properties or toxicological implications. In addition, certain catechins undergo epimerization during storage or thermal degradation, altering their absorption and metabolism [37], therefore, a robust method is necessary to properly characterize these catechins and their epimers. Some previous studies have described analytical methods for the determination and characterization of green tea catechins and related compounds. Rha et al. [31] developed a HPLC–UV-MS method to separate 19 phenolic compounds, including the eight catechins studied here. Ultra-performance liquid chromatography (UPLC) coupled with quadrupole-time-of-flight was used to characterize flavan-3-ols (C, EC, GC, EGC, etc.) and proanthocyanidins [37], and LC-MS have also been employed in previous studies to monitor the stability of catechins compounds [38]; however, previous high-resolution MS/MS analyses are limited [39].

### 4.1. Metabolism of Green Tea Catechins

EGCG is the most bioactive compound known in green tea, responsible for 32% of its antioxidant potential, while other catechins comprise only 5–12% [40]. This makes EGCG the most-studied green tea catechin. However, its analogs also have demonstrated important biological effects and sometimes greater stability, hence the necessity of describing their metabolism to better understand their impact in the human body and the effect of the structural differences on their biotransformation.

Under in vitro oxidative conditions, EGCG, its analogs and GA did not form any hydroxylated metabolites. This could be explained by their highly hydroxylated structures and the fact that they have been proven to inhibit NADPH oxidase activation by reducing O_2_^∙−^ production and thus protecting against peroxynitrite formation from NO [40,41].

When SAM was added to the incubation mixture, all catechins and GA formed mono-methylated and di-methylated metabolites (Figure 4 and Table 1). Methylation is a major biotransformation pathway for catechins [26,28], and has been described previously from in vitro and in animal studies [42] with the formation of 4′-O-methyl-EGC, 4″-O-methyl-EGCG, and 4″-O-methyl-EC being previously reported [28].

Although methylation is the primary metabolic pathway of these natural antioxidants, there are contradictory opinions in the literature about the role of methylation; with some considering that methylation can decrease the bioactivities, while others stating methylation improves the effectiveness of green tea catechins. For instance, Steffen et al. [43] indicated that methylation of EC at the 3′ or 4′ positions suppresses the superoxide (O_2_^∙−^) scavenging ability of the parent compound. Conversely, the major methylated tea catechins in oolong tea (MeEGCG and MeECG) demonstrated increased anti-adipogenicity activity compared to EGCG and ECG [44]. Also, the Japanese green tea (*Camellia sinensis* L.) cultivar “Benifuuki”, naturally rich with 3″-MeEGCG and 3″-MeECG, exhibits anti-allergic effects [45,46]. Maeda-Yamamoto et al. [45] demonstrated a higher potential of methylated catechins to inhibit histamine release than the non-methylated analogs. Given this contradiction in the literature, the effect of methylation of these molecules necessitates further study.

In the presence of NADPH and GSH, reactive quinone forms of catechins can react with glutathione. Sang et al. [47] found that EGCG can also be oxidized by peroxidase and hydrogen peroxide and then react with cysteine or glutathione, however, studies involving the formation GSH adducts of green tea catechins are still limited. The formation of such adducts scavenges potentially toxic reactive metabolites and reduces their potential for binding to cellular macromolecules [48,49]. From the results presented here, GSH was proven to bind on EGCG and its analogs in the B-ring or D-rings (Figure 6). Until now, it has been difficult to understand whether the broad bioactivities of green tea catechins are due to its antioxidant activity or to its interactions with specific molecular targets [50,51].

When both SAM and GSH were added to incubations, methylated mono GSH adducts were formed (Table 1). The ability of tea catechins to oxidize is negated by COMT-mediated methylation due to masking of the catechol moiety, however, conjugation of quinones with GSH does not eliminate their reactivity to COMT [52]. This also explains why di-methylated metabolites are no longer detected under these conditions. The competition between *O*-methylation and oxidation coupled to GSH conjugation of green tea catechins may have important implications in vivo; therefore, characterizing these novel metabolites is significant.

### 4.2. Metabolism of Alkyl Gallates

GA and analogs are used as potent antioxidants in various applications; however, their metabolism has not been extensively studied until now. It has been reported that with increased hydrophobicity comes significant increased cytotoxicity, but also improved antioxidant activity [53]. The more hydrophobic gallate esters (BG, OG, and LG) formed oxidative metabolites, unlike EG and PG (Table 2) with the oxidation occurring in the alkyl side chain. This could be explained by the specificity of microsomal cytochrome P450 enzymes, which is controlled by the lipophilicity of the substrate [54]. OG and LG can further oxidize to the corresponding aldehyde and carboxylic acids, a common reaction in the biotransformation of xenobiotics catalyzed by cytochromes P450 [55]. Methylation of gallate esters occurs on the hydroxy groups of galloyl moiety catalyzed by COMT [56,57].

All studied synthetic gallate esters formed several GSH adducts, in addition to different combinations of oxidation and methylation. Like catechins, synthetic gallates also produced methylated GSH adducts. Additionally, OG and LG produced hydroxylated GSH adducts (+O-2H+GSH). Di-glutathione adducts were also observed for PG and BG.

## 5. Conclusions

By studying the human in vitro metabolism of eight naturally occurring catechins, five synthetic gallate esters, and gallic acid, this study was able to compare the effect of subtle structural differences on metabolism. A single chromatographic method was developed for the analysis of all parent compounds and their numerous biotransformation products, with high-resolution tandem mass spectrometry enabling accurate mass measurements for elucidating their structures (Tables S1 and S2). The only exception was that for gallic acid metabolites, a slower gradient was used to enable retention of highly polar species. HRMS/MS was used to differentiate between many isomeric structures. Several new metabolites have been described for the first time in this report, particularly those of synthetic gallates. The formation of glutathione conjugates of methylated metabolites of green tea catechins and synthetic galloylated compounds is a novel biotransformation pathway not previously described for these molecules. A total of 116 metabolites were characterized in this work, while pinpointing which moieties are susceptible to oxidation, methylation, and glutathione conjugation (Figure 7 and Figure 8). The compilation of these results serves to increase our knowledge of different metabolic pathways potentially implicated in vivo when studying the effect of these compounds separately and in combination. Of course, the biological relevance of these metabolites should be further studied in vivo.

## Figures and Tables

**Table 1:** Summary of detected metabolites from studied natural antioxidants.

Biotransformation	Formula	RT (min)	*m/z* (ppm)	RT (min)	*m/z* (ppm)
**C/EC**	**C**			**EC**	
Parent	C_15_H_14_O_6_	6.5	289.0722 (1.5)	8.8	289.0720 (0.8)
+CH_2_	C_16_H_16_O_6_	10.1	303.0879 (1.6)	11.6	303.0877 (1)
		11.3	303.0878 (1.3)	13.0	303.0876 (0.6)
+2CH_2_	C_17_H_18_O_6_	13.0	317.1030 (−0.2)	14.1	317.1032 (0.4)
−2H+GSH	C_25_H_29_N_3_O_12_S	5.8	594.1403 (0.6)	5.1	594.1400 (0.1)
		6.7	594.1400 (0.1)	8.7	594.1404 (0.8)
+CH_2_-2H+GSH	C_26_H_31_N_3_O_12_S	7.4	608.1531 (−4.1)	9.0	608.1548 (−1.3)
		8.0	608.1554 (−0.3)	10.1	608.1549 (−1.1)
**GC/EGC**	**GC**			**EGC**	
Parent	C_15_H_14_O_7_	3.8	305.0673 (2)	6.0	305.0669 (0.7)
+CH_2_	C_16_H_16_O_7_	6.9	319.0829 (1.8)	9.1	319.0829 (1.8)
		7.8	319.0835 (3.7)	9.8	319.0827 (1.2)
+2CH_2_	C_17_H_18_O_7_	10.9	333.0989 (2.8)	13.2	333.0985 (1.6)
		11.9	333.0979 (−0.2)	11.5	333.0983 (−1.1)
−2H+GSH	C_25_H_29_N_3_O_13_S	2.5	610.1346 (−0.4)	4.6	610.1342 (-1)
		3.4	610.1350 (0.3)	5.8	610.1345 (−0.5)
−4H+2GSH	C_35_H_44_N_6_O_19_S_2_	3.6	457.0982 * (0.7)	5.5	457.0983 * (1)
+CH_2_-2H+GSH	C_26_H_31_N_3_O_13_S	5.9	624.1509 (0.7)	7.1	624.1501 (−0.6)
		6.9	624.1498 (−1.1)	7.9	624.1503 (−0.3)
**CG/ECG**	**CG**			**ECG**	
Parent	C_22_H_18_O_10_	11.9	441.0824 (−0.7)	11.7	441.0824 (−0.7)
+CH_2_	C_23_H_20_O_10_	14.1	455.0988 (0.9)	12.7	455.0978 (1.1)
		14.6	455.0983 (−0.2)	13.3	455.0985 (−0.3)
+2CH_2_	C_24_H_22_O_10_	16.4	469.1142 (0.4)	15.7	469.1143 (−0.6)
−2H+GSH	C_32_H_33_N_3_O_16_S	10.4	746.1476 (−4.4)	8.4	746.1514 (0.7)
		11.1	746.1504 (−0.6)	10.1	746.1512 (0.4)
		11.4	746.1491 (-2.4)	11.2	746.1514 (0.7)
−4H+2GSH	C_42_H_48_N_6_O_22_S_2_	10.7	525.1049 * (−1.9)	10.0	525.1061 * (0.4)
+CH_2_-2H+GSH	C_33_H_35_N_3_O_16_S	11.5	760.1648 (−2.3)	11.3	760.1672 (0.9)
		11.9	760.1633 (−4.2)	11.7	760.1670 (0.6)
**GCG/EGCG**	**GCG**			**EGCG**	
Parent	C_22_H_18_O_11_	9.9	457.0775 (−0.3)	9.3	457.0772 (−1)
+CH_2_	C_23_H_20_O_11_	12.3	471.0937 (0.9)	11.2	471.0929 (−0.8)
		12.7	471.0937 (0.9)	11.8	471.0938 (1.1)
+2CH_2_	C_24_H_22_O_11_	14.2	485.1091 (0.3)	13.5	485.1085 (−0.9)
		14.8	485.1094 (0.9)	14.6	485.1093 (0.7)
−2H+GSH	C_32_H_33_N_3_O_17_S	7.9	762.1434 (−3.1)	7.6	762.1451 (−0.9)
		8.6	762.1432 (−3.4)	8.8	762.1469 (1.5)
−4H+2GSH	C_42_H_48_N_6_O_23_S_2_	6.6	533.1026 * (−1.4)	7.3	533.1033 * (−0.1)
+CH_2_-2H+GSH	C_33_H_35_N_3_O_17_S	9.6	776.1592 (−2.9)	9.5	776.1610 (−0.6)
		10.2	776.1606 (−1.1)	10.2	776.1607 (−1)

*: doubly charged ion [M-2H]^2−^.

**Table 2:** Summary of detected metabolites from gallic acid and gallate ester analogs.

Biotransformation	Formula	RT (min)	*m/z* (ppm)
**GA ^a^ (Parent)**	C_7_H_6_O_5_	3.5	169.0142 (−0.3)
+CH_2_	C_8_H_8_O_5_	7.2	183.0302 (1.6)
		6.5	183.0298 (−0.5)
+2CH_2_	C_9_H_10_O_5_	11.3	197.0459 (1.8)
−2H+GSH	C_17_H_21_N_3_O_11_S	3.1	474.0828 (0.8)
+CH_2_-2H+GSH	C_18_H_23_N_3_O_11_S	4.2	488.0980 (−0.1)
**EG (Parent)**	C_9_H_10_O_5_	9.8	197.0460 (−2.4)
+CH_2_	C_10_H_12_O_5_	13.2	211.0617 (−2.7)
		14.4	211.0620 (−3.9)
−2H+GSH	C_19_H_25_N_3_O_11_S	8.1	502.1133 (0.7)
+CH_2_-2H+GSH	C_20_H_27_N_3_O_11_S	10.4	516.1296 (−0.6)
		11.5	516.1298 (−1)
**PG (Parent)**	C_10_H_12_O_5_	13.2	211.0612 (−0.2)
+CH_2_	C_11_H_14_O_5_	15.9	225.0770 (−0.9)
		16.9	225.0768 (−0.2)
−2H+GSH	C_20_H_27_N_3_O_11_S	10.9	516.1283 (1.9)
+CH_2_-2H+GSH	C_21_H_29_N_3_O_11_S	12.9	530.1452 (−0.5)
		13.9	530.1441 (1.5)
		14.4	530.1446 (0.7)
−4H+2GSH	C_30_H_42_N_6_O_17_S_2_	10.1	410.0951 * (−0.1)
**BG (Parent)**	C_11_H_14_O_5_	16.0	225.0775 (−3.2)
+CH_2_	C_12_H_16_O_5_	17.5	239.0928 (−1.6)
		17.8	239.0929 (−1.7)
+O	C_11_H_14_O_6_	8.4	241.0725 (−3.2)
		8.6	241.0724 (−3)
+O+CH_2_	C_12_H_16_O_6_	11.6	255.0880 (−2.4)
−2H+GSH	C_21_H_29_N_3_O_11_S	13.5	530.1436 (2.6)
+CH_2_-2H+GSH	C_22_H_31_N_3_O_11_S	15.2	544.1587 (3.5)
		16.2	544.1590 (3)
−4H+2GSH	C_31_H_44_N_6_O_17_S_2_	12.2	417.1032 * (−0.6)
**OG (Parent)**	C_15_H_22_O_5_	18.6	281.1394 (−0.1)
+CH_2_	C_16_H_24_O_5_	18.8	295.1544 (2.3)
		19.0	295.1544 (2.3)
+O	C_15_H_22_O_6_	17.1	297.1345 (−0.7)
		17.3	297.1340 (1)
+O+CH_2_	C_16_H_24_O_6_	17.8	311.1499 (0.4)
+2O	C_15_H_22_O_7_	14.2	313.1291 (−0.4)
OG aldehyde (+O-2H)	C_15_H_20_O_6_	17.4	295.1190 (−1)
OG acid (+2O-2H)	C_15_H_20_O_7_	17.2	311.1139 (−1)
−2H+GSH	C_25_H_37_N_3_O_11_S	18.1	586.2066 (1.7)
+CH_2_-2H+GSH	C_26_H_39_N_3_O_11_S	18.4	600.2228 (0.7)
+O-2H+GSH	C_25_H_37_N_3_O_12_S	15.5	602.2018 (1.1)
		15.1	602.2025 (0.4)
+O+CH_2_-2H+GSH	C_26_H_39_N_3_O_12_S	16.9	616.2180 (0.1)
**LG (Parent)**	C_19_H_30_O_5_	19.2	337.2017 (1)
+CH_2_	C_20_H_32_O_5_	19.3	351.2177 (−0.1)
		19.4	351.2172 (1.4)
+O	C_19_H_30_O_6_	18.5	353.1981 (3.5)
		18.7	353.1966 (0.9)
LG aldehyde (+O-2H)	C_19_H_28_O_6_	18.8	351.1811 (0.6)
LG acid (+2O-2H)	C_19_H_28_O_7_	18.6	367.1759 (0.7)
−2H+GSH	C_29_H_45_N_3_O_11_S	18.9	642.2707 (−0.8)
+CH_2_-2H+GSH	C_30_H_47_N_3_O_11_S	19.0	656.2857 (−1.4)
+O-2H+GSH	C_29_H_45_N_3_O_12_S	18.2	658.2657 (−0.9)

*: doubly charged ion [M-2H]^2−^; ^a^ GA incubations were analyzed with a slower gradient.

## Data Availability

Data are contained within the article and Supplementary Materials.

## Supplementary Materials

The following are available online at https://www.mdpi.com/article/10.3390/antiox11091635/s1, Figure S1. Overlaid extracted ion chromatograms of OG metabolites formed in HLM incubations. Peaks with asterisk (*) were increased by 10× for clarity, Table S1. LC-HRMS/MS data of detected metabolites from studied natural antioxidants, Table S2. LC-HRMS/MS data of gallates esters analogs metabolites.
