# Supplementary material for: Structural Elucidation of Novel Stable and Reactive Metabolites of Green Tea Catechins and Alkyl Gallates by LC-MS/MS
Source: Antioxidants (Basel). 2022 Aug 23;11(9):1635. doi: 10.3390/antiox11091635 (PMC9495999; doi:10.3390/antiox11091635)
Supplement: Supplementary file 1 [file antioxidants-11-01635-s001.zip › antioxidants-1854776-supplementary.pdf]

antioxidants

MDPI

Supplemental Material

Structural Elucidation of Novel Stable and Reactive Metabolites of Green Tea Catechins and Alkyl Gallates by LC-MS/MS

Ons Ousji and Lekha Sleno *

Figure S1. Overlaid extracted ion chromatograms of octyl gallate (OG) metabolites formed in HLM incubations

Table S1. LC-HRMS/MS data of detected metabolites from studied natural antioxidants

Table S2. LC-HRMS/MS data of ester gallates analogs metabolites

Antioxidants **2021**, *10*, 1635. https://doi.org/10.3390/antiox11091635 https://www.mdpi.com/journal/antioxidants

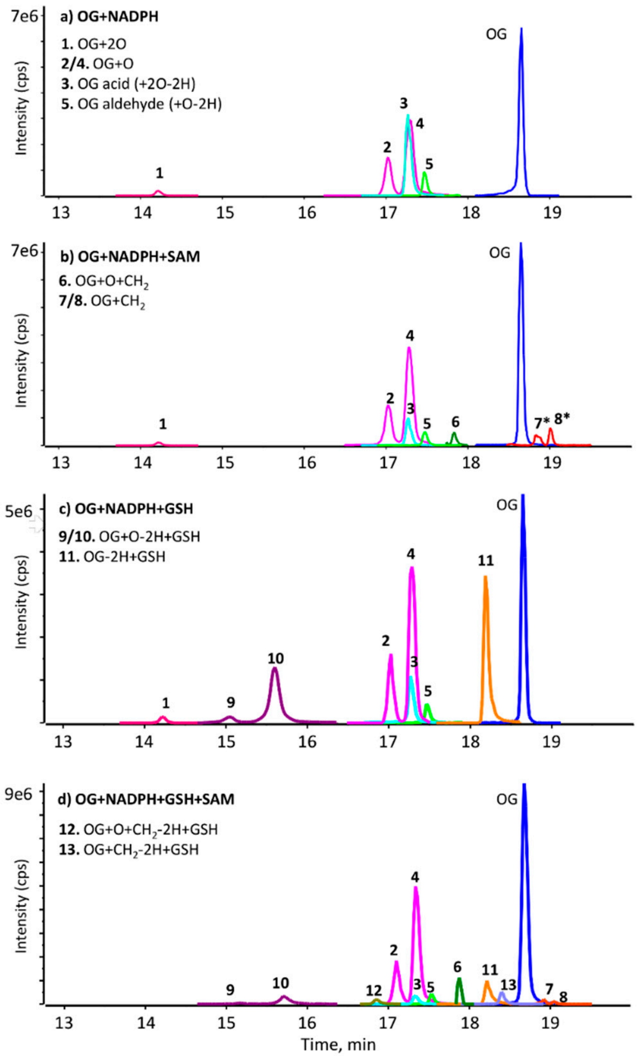

Figure S1. Overlaid extracted ion chromatograms of OG metabolites formed in HLM incubations. Peaks with asterisk (*) were increased by 10× for clarity.

Table S1. Summary of LC-HRMS/MS data of detected metabolites from studied natural antioxidants

Biotranformation	Formula	RT (min)	Measured *m/z* [M-H]- (ppm)	Selected MS/MS fragments
GA (Parent)	$C_7H_6O_5$	3.5	169.0142 (-0.3)	**125.0237**, **124.0163**, 107.0131, 97.0286, 81.0338, **79.0185**, 69.0341, 51.0235
+CH_2	$C_8H_8O_5$	7.2	183.0302 (1.6)	**168.0058**, 139.0389, **124.0156, 123.0081**, 95.0131
	$C_8H_8O_5$	6.5	183.0298 (-0.5)	**168.0057**, 139.0374, **124.0156, 123.0079**, 95.0130
+2CH_2	$C_9H_{10}O_5$	11.3	197.0459 (1.8)	**182.0220**, 166.9983, **153.0558**, **138.0331**, **123.0088**, 95.0135, 79.0179, 51.0239
-2H+GSH	$C_{17}H_{21}N_3O_{11}S$	3.1	474.0828 (0.8)	430.0899, 306.0769, **272.0890, 200.9861, 182.9746**, 166.9987, 123.0077
-CH_2-2H+GSH	$C_{18}H_{23}N_3O_{11}S$	4.2	488.0980 (-0.1)	272.0867, 215.0024, 199.9783, 155.9885, 111.0198
C (Parent)	$C_{15}H_{14}O_6$	6.5	289.0722 (1.5)	**245.0820, 221.0828, 205.0503, 203.0712,** 188.0475, **187.0401, 179.0347**, 165.0191, 161.0600, **151.0397,** 149.0243, **137.0242, 125.0239, 123.0447**, 121.0291, **109.0287**
+CH_2	$C_{16}H_{16}O_6$	10.1	303.0879 (1.6)	**285.0774, 270.0542**, 259.0979, **244.0746**, 235.0982, 220.0748, 219.0666, 202.0641, 165.0556, **151.0404, 137.0248**, 125.0245, **122.0376,** 109.0297, **97.0297**
	$C_{16}H_{16}O_6$	11.3	303.0878 (1.3)	270.0537, **244.0745**, 220.0741, 202.0636, **165.0563**, **150.0317**, **151.0402, 137.0249**, 125.0241, **122.0376,** 109.0299
+2CH_2	$C_{17}H_{18}O_6$	13.0	317.1030 (-0.2)	**299.0919,** 273.1113, **258.0893, 234.0905**, 219.0669, **179.0357, 165.0569, 151.0396, 123.0462, 122.0380, 111.0450,** 97.0294
-2H+GSH	$C_{25}H_{29}N_3O_{12}S$	5.8	594.1403 (0.6)	456.1082, **321.0440**, 303.0337, **277.0535**, 272.0889, 254.0802, 210.0886, 183.0125, 167.0362, 137.0232, 128.0353
	$C_{25}H_{29}N_3O_{12}S$	6.7	594.1400 (0.1)	456.1081, 414.0055, **321.0441, 272.0895, 254.0795**, 210.0895, 179.0464, **143.0471**, 128.0364
+CH_2-2H+GSH	$C_{26}H_{31}N_3O_{12}S$	7.4	608.1531 (-4.1)	**306.0764**, 301.0722, 288.0668, **272.0890**, 254.0812, 210.0885, 177.0358, 160.0072, 143.0464, 128.0352
	$C_{26}H_{31}N_3O_{12}S$	8.0	608.1554 (-0.3)	**306.0763**, 301.0720, 288.0664, **272.0883**, 254.0786, 210.0897, 177.0335, 160.0072, 143.0459, 128.0353
EC (Parent)	$C_{15}H_{14}O_6$	8.8	289.0720 (0.8)	**245.0807, 221.0815, 205.0499, 203.0706,** 188.0472, **187.0399, 179.0342**, 165.0187, 161.0596, **151.0394,** 149.0239, **137.0239, 125.0236, 123.0445**, 121.0291, **109.0285**
+CH_2	$C_{16}H_{16}O_6$	11.6	303.0877 (1.0)	**285.0774, 270.0542**, 259.0979, **244.0746**, 235.0982, 220.0748, 219.0666, 202.0641, 165.0556, **151.0404, 137.0248**, 125.0245, **122.0376,** 109.0297, **97.0297**
	$C_{16}H_{16}O_6$	13.0	303.0876 (0.6)	270.0524, **244.0731**, 220.0735, 202. 0625, **165.0545**, **150.0320**, 151.0391**,** 138.0307, **137.0240**, **125.0240**, **122.0376** 109.0299, 83.0140
+2CH_2	$C_{17}H_{18}O_6$	14.1	317.1032 (0.4)	**299.0921**, 284.0713, 273.1138, 258.0890, **234.9763**, 219.0673, **179.0356**, 175.9624, 165.0544, 152.0471, 151.0398, **123.0451, 122.0366,** 111.0454
-2H+GSH	$C_{25}H_{29}N_3O_{12}S$	5.1	594.1400 (0.1)	398.0152, **321.0445,** 303.0334, **277.0561**, 272.0895, 254.0784, 210.0908, 183.0132, 143.0469, 125.0270
	$C_{25}H_{29}N_3O_{12}S$	8.7	594.1404 (0.8)	456.1086, **321.0442, 272.0891**, 254.0790, 143.0464, 128.0357
+CH_2-2H+GSH	$C_{26}H_{31}N_3O_{12}S$	9.0	608.1548 (-1.3)	**306.0750**, 301.0708, 288.0640, **272.0877**, 254.0762, 210.0873, 177.0326, 160.0051, 143.0444, 128.0346
	$C_{26}H_{31}N_3O_{12}S$	10.1	608.1549 (-1.1)	**306.0753**, 301.0709, 288.0656, **272.0878**, 160.0070, 143.0463, 128.0344

Table S1. Summary of LC-HRMS/MS data of detected metabolites from studied natural antioxidants (continued)

Biotranformation	Formula	RT (min)	Measured *m/z* [M-H]⁻ (ppm)	Selected MS/MS fragments
GC (Parent)	$C_{15}H_{14}O_7$	3.8	305.0673 (-2)	261.0774, 221.0461, 219.0669, 179.0355, **167.0355, 165.0197**, 164.0120, **139.0403**, **137.0247**, **125.0243**, 111.0452
+CH2	$C_{16}H_{16}O_7$	6.9	319.0829 (-1.8)	301.0726, 260.0693, 236.0693, 218.0587, **181.0511**, 179.0355, **166.0270,** 165.0197, 138.0319, 137.0243, **125.0244**, 109.0291
	$C_{16}H_{16}O_7$	7.8	319.0835 (-3.7)	**275.0927**, **260.0689,** 251.0927, 243.0663, **236.0689, 235.0611, 233.0819, 220.0377, 218.0582, 217.0508**, **203.0352, 181.0501, 179.0349, 175.0400, 165.0188, 151.0960, 139.0397, 138.0318, 137.0240, 125.0239, 124.0161**
+2CH2	$C_{17}H_{18}O_7$	10.9	333.0989 (-2.8)	315.0883, 300.0650, 285.0415, 274.0852, 259.0617, 250.0850, 232.0745, 217.0513, **195.0664, 180.0432, 165.0197, 151.0402, 138.0317, 137.0243**, 125.0243, 109.0293
	$C_{17}H_{18}O_7$	11.9	333.0979 (0.2)	**315.0892,** 300.0645, **285.0412, 274.0851, 259.0619, 250.0851, 235.0620,** 217.0507, 195.0654, 180.0425**, 165.0197, 150.0421, 138.0317, 137.0241**, 125.0245, 111.0199, **97.0286**
-2H+GSH	$C_{25}H_{29}N_3O_{13}S$	2.5	610.1346 (0.4)	472.1066, **337.0409**, **306.0791**, 303.0544, **272.0905, 254.0806, 177.0211**, 143.0475, 128.0363, **125.0253**
	$C_{25}H_{29}N_3O_{13}S$	3.4	610.1350 (-0.3)	472.1058, **337.0400**, 306.0782, 303.0531, **272.0904**, 254.0797, 177.0207, 143.0471, 137.0253, 125.0251
+CH2-2H+GSH	$C_{26}H_{31}N_3O_{13}S$	5.9	624.1509 (-0.7)	317.0685, **306.0786**, 272.0895, 254.0794, 231.0674, 143.0462, 128.0360
	$C_{26}H_{31}N_3O_{13}S$	6.9	624.1498 (1.1)	486.1208, **351.0543**, 333.0457, **306.0768, 272.0883**, **254.0781**, 213.0239, 210.0890, 195.0101, 179.0420, **143.0459, 137.0240, 128.0356**, 125.0247
-4H+2GSH	$C_{35}H_{44}N_6O_{19}S_2$	3.6	457.0982*(-0.7)	**642.1091, 457.1009**, 369.0130, **335.0235, 306.0777**, **272.0907, 254.0788, 210.0893, 208.9927**, 196.9930, **179.0476, 143.0465, 137.0243, 128.0353**
EGC (Parent)	$C_{15}H_{14}O_7$	6.0	305.0669 (0.7)	261.0765, 221.0456, **219.0662**, 204.0432, **179.0350, 167.0352, 165.0195**, 164.0120, **139.0403, 137.0246**, 111.0454, 109.0300
+CH2	$C_{16}H_{16}O_7$	9.1	319.0829 (1.8)	**275.0927, 260.0696**, 243.0648, **236.0697**, 235.0611, 233.0816, 220.0378, **218.0585, 217.0511**, 203.0360, 181.0512, **179.0357, 166.0275, 165.0198, 139.0405, 138.0326, 137.0247, 125.0246, 124.0172**, 97.0289
	$C_{16}H_{16}O_7$	9.8	319.0827 (1.2)	260.0687, 218.0582, 181.0505, **166.0269**, **165.0193, 138.0322, 137.0243**, 125.0244
+2CH2	$C_{17}H_{18}O_7$	13.2	333.0985 (1.6)	259.0614, 195.0658, 180.0427, **165.0194,** 152.0480, 138.0316, **137.0246**, 125.0242
	$C_{17}H_{18}O_7$	11.5	333.0983 (-1.1)	193.0494, 181.0500, **179.0359, 166.0273,** 152.0469, **151.0397**, 137.0238, **138.0322**, 123.0453
-2H+GSH	$C_{25}H_{29}N_3O_{13}S$	4.6	610.1342 (-1)	472.1037, **337.0385**, 306.0771, **272.0891**, 254.0787, 210.0888, 177.0200, 143.0466, 137.0248, 128.0356, 125.0248
	$C_{25}H_{29}N_3O_{13}S$	5.8	610.1345 (-0.5)	481.0873, 442.0908, **337.0393**, **272.0895**, 254.0780, 210.0891, 179.0454, 177.0203, 125.0244
+CH2-2H+GSH	$C_{26}H_{31}N_3O_{13}S$	7.1	624.1501 (-0.6)	317.0668, **306.0769**, 288.0670, 272.0889, 254.0784, 231.0659, 160.0068, 143.0462, 128.0357
	$C_{26}H_{31}N_3O_{13}S$	7.9	624.1503 (-0.3)	317.0670, **306.0764**, 288.0656, 272.0889, 254.0776, 231.0656, 160.0073, 143.0461, 128.0353
-4H+2GSH	$C_{35}H_{44}N_6O_{19}S_2$	5.5	457.0983*(1)	642.1082, 457.0991, 369.0109, **335.0248, 306.0782**, **272.0899**, **254.0793, 208.9925, 196.9923, 179.0473, 143.0471, 137.0253**, 128.0361, 125.0248

Table S1. Summary of LC-HRMS/MS data of detected metabolites from studied natural antioxidants (continued)

Biotranformation	Formula	RT (min)	Measured *m/z* [M-H]- (ppm)	Selected MS/MS fragments
CG (Parent)	$C_{22}H_{18}O_{10}$	11.9	441.0824 (-0.7)	331.0454, 303.0508, **289.0712**, **271.0611, 245.0816**, 205.0506, 203.0719, **193.0147**, **169.0143, 137.0249, 125.0244, 124.0170**
+CH2	$C_{23}H_{20}O_{10}$	14.1	455.0988 (0.9)	411.1077, 317.0667, **305.0670**, 290.0439, **271.0611, 253.0512**, 227.0716, **183.0305, 168.0070**, 159.0455, **145.0298, 137.0246, 125.0245**
	$C_{23}H_{20}O_{10}$	14.6	455.0983 (-0.2)	**303.0876**, 285.0773, 270.0537, **183.0302, 169.0145**, 137.0246, **125.0244, 124.0163**
+2CH2	$C_{24}H_{22}O_{10}$	16.4	469.1142 (0.4)	**305.0675, 303.0869**, **285.0774**, **270.0533**, 246.0536, **241.0869,** 226.035, **183.0297**, 165.0198, 149.0245, 137.0245, 124.0157
-2H+GSH	$C_{32}H_{33}N_3O_{16}S$	10.4	746.1476 (-4.4)	**576.1255, 473.0506, 321.0426, 303.0320, 272.0877,** 254.0774**, 169.0140,** 143.0458
	$C_{32}H_{33}N_3O_{16}S$	11.1	746.1504 (-0.6)	617.1034, 608.1144, 594.1350, **473.0500,** 335.0216**, 321.0426,** 303.0329**, 272.0876,** 254.0776**, 169.0138,** 143.0457, 125.0243
	$C_{32}H_{33}N_3O_{16}S$	11.4	746.1491 (-2.4)	**474.0791, 473.0500, 439.0642, 306.0747, 272.0875**, 254.0778, **200.9853, 182.9754**, 143.0463
-4H+2GSH	$C_{42}H_{48}N_6O_{22}S_2$	10.7	525.1049* (-1.9)	**779.1352, 778.1167**, 744.0863**, 525.1016, 505.0230**, 471.0357, 472.0620, **306.0751, 289.0700, 272.0876, 254.0765**, 210.0872, **179.0452, 143.0459, 128.0349, 125.0240**
+CH2-2H+GSH	$C_{33}H_{35}N_3O_{16}S$	11.5	760.1648 (-2.3)	**453.0806, 306.0760, 301.0711**, 254.0778, 143.0465, 128.0352
	$C_{33}H_{35}N_3O_{16}S$	11.9	760.1633 (-4.2)	**453.0802, 306.0753, 301.0706**, 272.0876, 254.0768, 215.0706, 143.0462
ECG (Parent)	$C_{22}H_{18}O_{10}$	11.7	441.0824 (-0.7)	331.0441, 303.0514, **289.0724**, 271.0606, 245.0823, 205.0509, 203.0716, 193.0145, **169.0144,** 137.0243, 125.0244**, 124.0168**
+CH2	$C_{23}H_{20}O_{10}$	12.7	455.0978 (1.1)	217.0663, **305;0661, 271.0605, 253.0504**, 227.0715, **183.0298, 145.0293, 137.0241**, **125.0239**, 124.0166
	$C_{23}H_{20}O_{10}$	13.3	455.0985 (-0.6)	305.0658, **289.0714**, 271.0612, 245.0816, 203.0714, **183.0298**, 168.0064, 125.0239
+2CH2	$C_{24}H_{22}O_{10}$	15.7	469.1143 (-0.3)	**305.0669, 303.0889, 289.0735, 285.0765, 270.0544**, 246.0526, 241.0894, **183.0297,** 168.0064, 149.0236, **137.0241**
-2H+GSH	$C_{32}H_{33}N_3O_{16}S$	8.4	746.1514 (0.7)	576.1310, **473.0547**, **321.0444, 303.0337, 272.0892**, 254.0785, **169.0143**, 143.0459
	$C_{32}H_{33}N_3O_{16}S$	10.1	746.1512 (0.4)	594.1399, **473.0532**, 321.0438**, 272.0887**, 169.0143
	$C_{32}H_{33}N_3O_{16}S$	11.2	746.1514 (0.7)	**474.0806, 473.0543**, 439.0679, **306.0773,** 289.0726, **272.0894**, **200.9866**, 182.9764
-4H+2GSH	$C_{42}H_{48}N_6O_{22}S_2$	10.0	525.1061*(0.4)	**525.1081, 506.0516, 505.0263,** 488.0462, 471.0403, 462.0665, **306.0770, 289.0731, 272.0897, 254.0781**, 232.9588, **214.9481,** 210.0893, **179.0461, 143.0466, 128.0356, 125.0251**
+CH2-2H+GSH	$C_{33}H_{35}N_3O_{16}S$	11.3	760.1672 (0.9)	**453.0819, 306.0773, 301.0730**, 272.0888, 254.0781, 143.0462
	$C_{33}H_{35}N_3O_{16}S$	11.7	760.1670 (0.6)	**453.0829, 306.0773, 301.0725**, 272.0894, 254.0769, 143.0458

Table S1. Summary of LC-HRMS/MS data of detected metabolites from studied natural antioxidants (continued)

Biotranformation	Formula	RT (min)	Measured *m/z* [M-H]$^-$ (ppm)	Selected MS/MS fragments
GCG (Parent)	$C_{22}H_{18}O_{11}$	9.9	457.0775 (-0.3)	331.0465, 305.0670, 287.0560, 269.0467, 219.0662, 193.0662, **169.0144**, 161.0246, 137.0244, **125.0244**
+CH2	$C_{23}H_{20}O_{11}$	12.3	471.0937 (0.9)	333.0625, **287.0568,** 269.0461, 243.0668, **183.0307**, 169.0147, 137.0249, **125.0246**
	$C_{23}H_{20}O_{11}$	12.7	471.0937 (0.9)	**319.0828, 305.0671, 287.0568**, 269.0459, **183.0300, 169.0143, 161.0245**, 137.0245, **125.0243**, 124.0166
+2CH2	$C_{24}H_{22}O_{11}$	14.2	485.1091 (0.3)	**301.0724**, **283.0619, 268.0384**, 257.0817, **242.0481, 215.0715**, 213.0559, **183.0311, 168.0067**, 139.0396, **125.0246, 124.0168**
	$C_{24}H_{22}O_{11}$	14.8	485.1094 (0.9)	441.1175, **305.0669, 301.0719**, 290.0451, **269.0457**, **251.0349, 225.0559,** 183.0301, 168.0065, 137.0242, 125.0244, 124.0170
-2H+GSH	$C_{32}H_{33}N_3O_{17}S$	7.9	762.1434 (-3.1)	**592.1192, 574.1104, 489.0456**, 337.0377, **319.0256, 306.0742, 272.0865, 254.0765**, 210.0874, 179.0456, **169.0130, 143.0458, 128.0348**
	$C_{32}H_{33}N_3O_{17}S$	8.6	762.1432 (-3.4)	**489.0475, 474.0795, 455.0604**, 430.0906, **306.0756, 272.0879, 200.9858**, 182.9756
-4H+2GSH	$C_{42}H_{48}N_6O_{23}S_2$	6.6	533.1026* (-1.4)	794.1126, 624.0934, 590.1053, **533.0994**, 498.0617, 350.9976, **317.0115, 306.0753**, 272.0876, **254.0771**, 210.0874, 179.0451, **169.0132**, **143.0455, 125.0238, 128.0346**
+CH2-2H+GSH	$C_{33}H_{35}N_3O_{17}S$	9.6	776.1592 (-2.9)	**471.0802, 470.0784, 469.0735, 319.0708, 318.0696, 317.0656, 308.0721, 307.0782, 306.0747**, 299.0540, 273.0769, 272.0871, 231.0656, 167.0345, 143.0451, **137.0240**, 128.0342, 125.0225
	$C_{33}H_{35}N_3O_{17}S$	10.2	776.1606 (-1.1)	**471.0803, 306.0774, 301.0725**, 272.0893, 254.0768, 143.0457
EGCG (Parent)	$C_{22}H_{18}O_{11}$	9.3	457.0772 (-1)	331.0453, **305.0666**, 287.0555, 269.0461, 193.0142, 179.0352, **169.0137**, 165.0188, **125.0241**
+CH2	$C_{23}H_{20}O_{11}$	11.2	471.0929 (-0.8)	333.0618, 319.0818, **169.0142**, 161.0242, 139.0398, **125.0240**
	$C_{23}H_{20}O_{11}$	11.8	471.0938 (1.1)	**305.0676, 287.0569**, **183.0306, 161.0249**, **125.0246**
+2CH2	$C_{24}H_{22}O_{11}$	13.5	485.1085 (-0.9)	**441.1190, 305.0661, 301.0712, 283.0600, 269.0454, 251.0380, 217.0025, 183.0302, 168.0059, 141.0171, 137.0249, 125.0241**
	$C_{24}H_{22}O_{11}$	14.6	485.1093 (0.7)	441.1176, **305.0670, 301.0719**, **269.0456**, **251.0349,** 183.0301, 168.0065, 137.0242, 125.0244, 124.0170
-2H+GSH	$C_{32}H_{33}N_3O_{17}S$	7.6	762.1451 (-0.9)	610.1336, **592.1232**, 574.1125, **489.0491**, 337.0407, **319.0286**, 306.0767, **272.0890,** 254.0783, 169.0142, 143.0463, 128.0349
	$C_{32}H_{33}N_3O_{17}S$	8.8	762.1469 (1.5)	592.1217, **489.0480, 474.0809**, 455.0605, 337.0368, 306.0764, 272.0889, **200.9862**, 182.9752
-4H+2GSH	$C_{42}H_{48}N_6O_{23}S_2$	7.3	533.1033*(-0.1)	897.1959, 794.1186, **624.0964, 533.1035,** 317.0130, 306.0770, **272.0891, 254.0789**, 210.0886, 179.0466, **169.0145**, 143.0460, **128.0354, 125.0242**
+CH2-2H+GSH	$C_{33}H_{35}N_3O_{17}S$	9.5	776.1610 (-0.6)	**469.0777, 317.0662, 306.0763**, 299.0558, 272.0882, 231.0651, 169.0137
	$C_{33}H_{35}N_3O_{17}S$	10.2	776.1607 (-1)	**469.0766, 317.0666, 306.0767, 299.0559,** 272.0881, 231.0653, 169.0135

*: doubly charged ions [M-2H]$^{2-}$; Fragment ions with >20% relative intensity compared to base peak are listed in bold, base peaks are underlined**.** GA incubations were injected with a slower gradient.

Table S2. Summary of LC-HRMS/MS data of ester gallates analogs metabolites

Biotransformation	Formula	RT (min)	Measured *m/z* [M-H]$^-$ (ppm)	Selected MS/MS fragments
EG (Parent)	$C_9H_{10}O_5$	9.8	197.0460 (-2.4)	**169.0136, 168.0061, 125.0231, 124.0161, 78.011**
+CH_2	$C_{10}H_{12}O_5$	13.2	211.0617 (-2.7)	197.0421, **196.0377,** 168.0047, **166.9987**, 139.0038, 124.0149, **123.0087**, 95.0137, 79.0188
	$C_{10}H_{12}O_5$	14.4	211.0620 (-3.9)	197.0410, **196.0376,** 168.0056, 183.0294, **166.9986**, 139.0040, 124.0144, **123.0088**
-2H+GSH	$C_{19}H_{25}N_3O_{11}S$	8.1	502.1133 (0.7)	**306.0769, 272.0882, 254.0782, 229.0178, 210.0883, 182.9761**, 179.0458, **143.0467, 128.0354**
+CH_2-2H+GSH	$C_{20}H_{27}N_3O_{11}S$	10.4	516.1296 (-0.6)	**272.0880,** 254.0787, **243.0328,** 228.0096, 210.0883, 179.0467, **143.0460, 128.0349**
	$C_{20}H_{27}N_3O_{11}S$	11.5	516.1298 (-1)	306.0751, **272.0889, 254.0782, 243.0348, 228.0106,** 210.0889, 196.9921, 179.0465, **143.0459, 128.0349**
PG (Parent)	$C_{10}H_{12}O_5$	13.2	211.0612 (-0.2)	**169.0139**, 168.0061, **125.0230**, **124.0159**, 78.0109
+CH_2	$C_{11}H_{14}O_5$	15.9	225.0770 (-0.9)	**211.0577,** 210.0536, 168.0048, **166.9991**, 139.0037, 124.0226, **123.0088**, 95.0139, 79.0190
	$C_{11}H_{14}O_5$	16.9	225.0768 (-0.2)	**211.0585,** 210.0544, 183.0300, 168.0057, **166.9995**, 139.0043, 124.0142, **123.0093**, 95.0139, 79.0193
-2H+GSH	$C_{20}H_{27}N_3O_{11}S$	10.9	516.1283 (1.9)	**306.0765, 272.0888, 254.0778, 243.0328,** 210.0880, **182.9756**, 179.0460, **143.0461, 128.0348**
-4H+2GSH	$C_{30}H_{42}N_6O_{17}S_2$	10.1	410.0951* (-0.1)	548.1019, 410.0965, **350.0382,** 328.0613, 304.0594, **272.0890**, **254.0778**, 241.0171, **214.9478, 210.0876**, 209.0441, **179.0456, 143.0457, 128.0350**
+CH_2-2H+GSH	$C_{21}H_{29}N_3O_{11}S$	12.9	530.1452 (-0.5)	306.0795, **273.0912, 272.0886, 257.0498, 254.0767,** 225.0246, 211.0929, 210.0909, **179.0460, 143.0461, 128.0351**
	$C_{21}H_{29}N_3O_{11}S$	13.9	530.1441 (1.5)	306.0766, **272.0884, 257.0490, 254.0775,** 225.0222, 210.0886, 179.0459, **143.0457, 128.0351**
	$C_{21}H_{29}N_3O_{11}S$	14.4	530.1446 (0.7)	448.0034, 306.0775, **272.0878, 257.0491, 254.0767**, 242.0263, 210.0877, 196.9906, 179.0447, **143.0462, 128.0354**
BG (Parent)	$C_{11}H_{14}O_5$	16.0	225.0775 (-3.2)	**169.0139, 168.0063, 125.0232, 124.0167, 78.0111**
+O	$C_{11}H_{14}O_6$	8.4	241.0725 (-3.2)	**169.0144,** 168.0065, **125.0239,** 124.0165, 78.0110, 59.0138
	$C_{11}H_{14}O_6$	8.6	241.0724 (-3)	**169.0139,** 168.0054, **125.0238,** 124.0163, 59.0137
+CH_2	$C_{12}H_{16}O_5$	17.5	239.0928 (-1.6)	225.0727, **224.0690, 166.9989**, 139.0050, 124.0140, **123.0088**, 95.0136, 79.0187
	$C_{12}H_{16}O_5$	17.8	239.0929 (-1.7)	**225.0713**, 224.0685, 183.0298, 168.0047, **166.9985**, 138.0326, 124.0146, **123.0087**, 95.0132, 79.0188
+O+CH_2	$C_{12}H_{16}O_6$	11.6	255.0880 (2.4)	241.0681, 2400644, 194.9384, 168.0045, 166.9985, 124.0144, 123.0090, 78.9582
-2H+GSH	$C_{21}H_{29}N_3O_{11}S$	13.5	530.1436 (2.6)	**306.0748, 272.0872, 257.0470, 254.0766**, 223.0616, **210.0879**, 197.0563, **182.9747, 179.0457,** 146.0456, **143.0454, 128.0347**
-4H+2GSH	$C_{31}H_{44}N_6O_{17}S_2$	12.2	417.1032* (-0.6)	**562.1161**, 417.1045, 304.0571, **289.0179, 272.0876, 255.0302, 254.0784,** 214.9475, 210.0867, **179.0462**, 146.0456, **143.0464, 128.0358**, 74.0249
+CH_2-2H+GSH	$C_{22}H_{31}N_3O_{11}S$	15.2	544.1587 (3.5)	462.0798, 380.0784, **272.0877, 271.0638, 256.0404,** 210.0886, 179.0466, **143.0463,** 128.0353, 99.0558, 74.0250
	$C_{22}H_{31}N_3O_{11}S$	16.2	544.1590 (3)	306.0767, **272.0886, 271.0631, 254.0789,** 225.0780, 210.0880, 196.9907, 179.0451, **143.0464, 128.0345**

Table S2. Summary of LC-HRMS/MS data of ester gallates analogs metabolites (continued)

Biotransformation	Formula	RT (min)	Measured *m/z* $[M-H]^-$ (ppm)	Selected MS/MS fragments
OG (Parent)	$C_{15}H_{22}O_5$	18.6	281.1394 (-0.1)	**169.0124, 168.0054**, 151.0035, 140.0110, **125.0221**, **124.0153**, 78.0105
+O	$C_{15}H_{22}O_6$	17.1	297.1345 (-0.7)	**279.1226, 269.1410, 253.1447, 235.1349, 169.0141**, 168.0063, **125.0235, 124.0160**
	$C_{15}H_{22}O_6$	17.3	297.1340 (1)	**279.1236, 269.1419, 253.1442, 235.1390, 169.0134, 168.0060**, 151.0034, **125.0227, 124.0154**, 78.0104
+2O	$C_{15}H_{22}O_7$	14.2	313.1291 (-0.4)	**169.0141,** 168.0063, **151.0039, 125.0237, 124.0163**
+O-2H (aldehyde)	$C_{15}H_{20}O_6$	17.4	295.1190 (-1)	267.1227, 251.1284, 237.0794, **169.0137,** 168.0056, **125.0232, 124.0156**
+2O-2H (acid)	$C_{15}H_{20}O_7$	17.2	311.1139 (-1)	**293.1024**, 267.1239, **169.0136, 159.1018, 151.0027, 125.0232, 124.0153, 123.0079**
+CH2	$C_{16}H_{24}O_5$	18.8	295.1544 (2.3)	281.2162, 280.1312, **271.2162**, 169.1234, 166.9988, 139.0034, 124.0163, 123.0086
	$C_{16}H_{24}O_5$	19.0	295.1544 (2.3)	281.1303, 280.1308, 277.2170, 251.2381, 197.1186, 169.1235, 166.9990, 139.0030, 124.0084
+O+CH2	$C_{16}H_{24}O_6$	17.8	311.1499 (0.4)	**296.1254**, 181.0131, **166.9979**, 124.0081, **123.0081**
-2H+GSH	$C_{25}H_{37}N_3O_{11}S$	18.1	586.2066 (1.7)	**313.1105, 306.0760, 272.0882, 254.0775, 210.0886, 182.9752, 179.0465, 143.0459, 128.0351**
+O-2H+GSH	$C_{25}H_{37}N_3O_{12}S$	15.5	602.2018 (1.1)	**329.1051, 272.0874, 254.0773**, 210.0881, **182.9754, 143.0459, 128.0351**
	$C_{25}H_{37}N_3O_{12}S$	15.1	602.2025 (0.4)	**329.1073, 306.0766, 272.0890, 254.0784,** 210.0873, **182.9762, 143.0454,** 128.0351
+CH2-2H+GSH	$C_{26}H_{39}N_3O_{11}S$	18.4	600.2228 (0.7)	**327.1270, 272.0878**, 254.0781, 210.0883, 179.0463, **143.0466,** 128.0358
+O+CH2-2H+GSH	$C_{26}H_{39}N_3O_{12}S$	16.9	616.2180 (0.1)	343.1223, **272.0883,** 254.0786, 210.0893, 143.0464, 128.0351
LG (Parent)	$C_{19}H_{30}O_5$	19.2	337.2017 (1)	**169.0124, 168.0051,** 151.0031, 140.0109, **125.0224, 124.0150**, 78.0106
+O	$C_{19}H_{30}O_6$	18.5	353.1981 (3.5)	335.2220, 223.1333, 169.0138, 168.0063, 125.0234, 124.0160
	$C_{19}H_{30}O_6$	18.7	353.1966 (0.9)	309.2060, **169.0139, 168.0062**, 151.0034, 140.0111, **125.0234, 124.0159**, 78.0108
+2O-2H (acid)	$C_{19}H_{28}O_7$	18.6	367.1759 (0.7)	349.1654, 323.1844, **215.1650**, 169.0141, **151.0035**, 125.0239, 124.0162, **123.0084**
+O-2H (aldehyde)	$C_{19}H_{28}O_6$	18.8	351.1811 (-0.6)	291.1927, **269.2106**, 169.0136, 124.0160
+CH2	$C_{20}H_{32}O_5$	19.3	351.2177 (-0.1)	337.1976, **336.1929**, 253.2166, 168.0052, **166.9981**, 152.0104, 139.0030, 124.0151, **123.0083**
	$C_{20}H_{32}O_5$	19.4	351.2172 (1.4)	337.1978, **336.1934**, 183.0300, 168.0054, **166.9984**, 152.0104, 139.0034, 124.0152, **123.0083**
-2H+GSH	$C_{29}H_{45}N_3O_{11}S$	18.9	642.2707 (-0.8)	**369.1723, 306.0757, 272.0879, 254.0775**, 210.0878, **182.9753, 143.0457,** 128.0346
+O-2H+GSH	$C_{29}H_{45}N_3O_{12}S$	18.2	658.2657 (-0.9)	**385.1664, 306.0748, 272.0875, 254.0765, 182.9749, 143.0456,** 128.0347
+CH2-2H+GSH	$C_{30}H_{47}N_3O_{11}S$	19.0	656.2857 (-1.4)	**383.1876,** 368.1650, **272.0874,** 254.0776, 210.0875, 179.0461, **143.0461,** 128.0354

*: doubly charged ions $[M-2H]^{2-}$; Fragment ions with >20% relative intensity compared to base peak are listed in bold, base peaks are underlined
